# Long‐term management changes topsoil and subsoil organic carbon and nitrogen dynamics in a temperate agricultural system

**DOI:** 10.1111/ejss.12359

**Published:** 2016-07-15

**Authors:** A. S. Gregory, J. A. J. Dungait, C. W. Watts, R. Bol, E. R. Dixon, R. P. White, A. P. Whitmore

**Affiliations:** ^1^ Department of Sustainable Soils and Grassland SystemsRothamsted ResearchHarpendenHertfordshire AL5 2JQUK; ^2^ Department of Sustainable Soils and Grassland SystemsRothamsted ResearchNorth Wyke, OkehamptonDevon EX20 2SBUK; ^3^ Terrestrial Biogeochemistry Group, Institute of Bio‐ and Geosciences IBG‐3: AgrosphereForschungszentrum Jülich GmbHJülich52425Germany; ^4^ Department of Computational and Systems BiologyRothamsted ResearchHarpendenHertfordshire AL5 2JQUK

## Abstract

Soil organic carbon (SOC) and nitrogen (N) contents are controlled partly by plant inputs that can be manipulated in agricultural systems. Although SOC and N pools occur mainly in the topsoil (upper 0.30 m), there are often substantial pools in the subsoil that are commonly assumed to be stable. We tested the hypothesis that contrasting long‐term management systems change the dynamics of SOC and N in the topsoil and subsoil (to 0.75 m) under temperate conditions. We used an established field experiment in the UK where control grassland was changed to arable (59 years before) and bare fallow (49 years before) systems. Losses of SOC and N were 65 and 61% under arable and 78 and 74% under fallow, respectively, in the upper 0.15 m when compared with the grass land soil, whereas at 0.3–0.6‐m depth losses under arable and fallow were 41 and 22% and 52 and 35%, respectively. The stable isotopes ^13^C and ^15^N showed the effects of different treatments. Concentrations of long‐chain n‐alkanes C_27_, C_29_ and C_31_ were greater in soil under grass than under arable and fallow. The dynamics of SOC and N changed in both topsoil and subsoil on a decadal time‐scale because of changes in the balance between inputs and turnover in perennial and annual systems. Isotopic and geochemical analyses suggested that fresh inputs and decomposition processes occur in the subsoil. There is a need to monitor and predict long‐term changes in soil properties in the whole soil profile if soil is to be managed sustainably.

**Highlights:**

- Land‐use change affects soil organic carbon and nitrogen, but usually the topsoil only is considered.
- Grassland cultivated to arable and fallow lost 13–78% SOC and N to 0.6 m depth within decades.
- Isotopic and biomarker analyses suggested changes in delivery and turnover of plant‐derived inputs.
- The full soil profile must be considered to assess soil quality and sustainability.

## Introduction

Carbon (C) stored in soil worldwide (1200–2400 Pg), typically in organic forms, exceeds that stored in the atmosphere (720–750 Pg) and terrestrial plants (550–835 Pg) combined (Batjes, 1996; Scharlemann *et al.*, 2014). Soil organic C (SOC) content is controlled predominantly by organic inputs from plants and soil type. Soil type (i.e. mineralogy and texture) is essentially ‘fixed’, whereas organic inputs can be manipulated by land use in managed systems. In agricultural systems, organic inputs are affected markedly by cultivation. The initial loss of SOC from arable cropping can be large and the return of C to soil relatively small from roots and unwanted crop residues in an annually‐harvested crop, compared with that under perennial systems (Wiesmeier *et al.*, 2012; Chapman *et al.*, 2013). Gregory *et al.* (2014) reported that the total SOC stocks in England and Wales under grassland and arable management were 1154 and 812 Tg (187 and 168 Mg ha^−1^ on an area basis), respectively. Changes in soil management lead to a change in both the SOC content and its equilibrium as it re‐adjusts to new levels of input and output. Typically, soil loses SOC faster than it gains it following management changes between arable and grassland systems (Johnston *et al.*, 2009; Poeplau *et al.*, 2011). Furthermore, modifications of soil structure in response to management have subsequent effects on SOC dynamics, for instance the potential for SOC to move down the soil profile in dissolved and colloid‐associated forms, processes that are more prevalent under grass than arable management (Baisden *et al.*, 2002; Kindler *et al.*, 2011). Most soil nitrogen (N) is predominantly found in organic compounds, even though inorganic nitrate and ammonium may be added in fertilizers or may occur naturally. Consequently, soil N is subject to similar processes to those of SOC and often closely follows the same patterns. The SOC:total N ratio in soil has a narrow range (around 9–14) in general across a range of soil types (Johnston *et al.*, 2009), although an increase in the proportion of ammonium N adsorbed in clayey subsoil minerals might weaken the link with SOC (Jenkinson *et al.*, 2008).

Most studies of SOC in agricultural soil focus on the topsoil (to 30‐cm depth) because it is the main zone of activity for crop roots, and where the need to understand nutrient and water use efficiency is paramount. Although considerable concentrations of SOC occur in the topsoil, there can be equal or greater total amounts in the subsoil (Jobbágy & Jackson, 2000; Gregory *et al.*, 2014), which can be an important component of the global C cycle (Baisden *et al.*, 2002). Recent interest in the subsoil has focused on its importance as a repository of SOC where the potential for increasing stocks has yet to be realized. Subsoil SOC was commonly assumed to be stable (Baisden & Parfitt, 2007; Rumpel & Kögel‐Knabner, 2011) and strongly affected by soil type (Meersmans *et al.*, 2009; Wiesmeier *et al.*, 2012). All SOC is now considered to be inherently unstable thermodynamically (Schmidt *et al.*, 2011), with little evidence of any difference in decomposability between topsoil and subsoil SOC (Fontaine *et al.*, 2007; Salomé *et al.*, 2010) or selective preservation of lignin or lipids (Amelung *et al.*, 2008; Marschner *et al.*, 2008). Any perceived stability in the subsoil or elsewhere might be linked to the soil physical environment rather than molecular recalcitrance (Bol *et al.*, 2009; Dungait *et al.*, 2012). The importance of the subsoil SOC pool is recognized increasingly, and the effects of soil management on SOC dynamics in the full profile are being explored (Don *et al.*, 2009; Schipper *et al.*, 2010; Wiesmeier *et al.*, 2012; Beniston *et al.*, 2014).

To understand the dynamics of subsoil SOC, comparisons of rigorous long‐term management experiments, where SOC inputs have been manipulated, need to be exploited. The Highfield ley‐arable long‐term experiment at Rothamsted Research (UK) provides an opportunity to study the effect of contrasting agricultural management systems on soil. The experiment has been used to examine the effects of management on topsoil SOC (Hirsch *et al.*, 2009; Johnston *et al.*, 2009), but not on that at depth in the profile. Our aim was to test the hypothesis that contrasting long‐term management systems change the dynamics of SOC and N in both the topsoil and subsoil. To address this, we measured not only bulk SOC and N, but also the stable isotopes ^13^C and ^15^N and plant‐derived long‐chain *n*‐alkanes, to explore SOC and N cycling *in situ*. Stable isotope ratios provide information on sources and processes of SOC, rates of transformation and environmental conditions (Amelung *et al.*, 2008), whereas long‐chain *n*‐alkanes are the most easily‐detectable components of lipids and are geochemical biomarkers of above‐ground plant inputs (van Bergen *et al.*, 1997).

## Materials and methods

### Experimental site and soil

**Figure 1:**
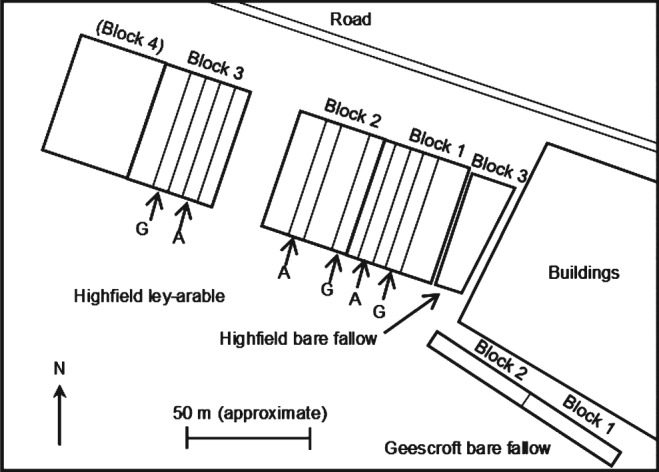
Plan of the field site showing the location of the ley‐arable and bare fallow experiments. Plots under long‐term grass (G) and arable (1949–; A) treatments in blocks 1–3 of the ley‐arable experiment, and the division of the long‐term bare fallow treatments (1959–) into three blocks are indicated.

The Highfield ley‐arable long‐term experiment was established at Rothamsted Research, Harpenden, UK (51.80 N 0.36 W), in 1949 in a field that had been under permanent grass since at least 1838 (Barré *et al.*, 2010), to assess the effects of different cropping systems on soil organic matter (SOM) and yields (Rothamsted Research, 2006). Six ley‐arable treatments were established in 50 m × 7 m plots in a randomized complete block design with four blocks. We focused on two treatments: permanent grass (predominantly rye grass, *Lolium perenne* L.) and continuous arable (winter wheat, *Triticum aestivum* L.). In 1959 a single trapezoidal area of grassland of ∼900 m^2^ adjacent to the ley‐arable experiment was ploughed. It has remained as permanent bare fallow by mouldboard ploughing and cultivating two to four times per year to 0.23 m depth (also the case with the arable treatment) (Barré *et al.*, 2010). A further adjacent narrow strip of land (approximately 100 m × 5 m) in Geescroft field, 30 m from one end of the bare fallow treatment, has had a similar long‐term management history. These two areas have provided a long‐term bare fallow treatment with the same soil type. The three treatments of interest are referred to hereafter as ‘grass’, ‘arable’ and ‘fallow’ for brevity and the field layout is depicted in Figure 1.

The site has an annual mean temperature (1992–2014) and rainfall (1981–2010) of 10.2°C and 718 mm, respectively (Scott *et al.*, 2014). The soil is a stagnogleyic paleo‐argillic brown earth (Batcombe series) (Avery, 1980) or a Chromic Luvisol (IUSS‐ISRIC‐FAO, 2006). It has a silty clay loam texture developed on recent clay‐with‐flints over Eocene London Clay and an original profile description from 1945 under long‐term grass is given in Table 1 (Avery & Catt, 1995).

**Table 1:** An original profile description from 1945 of the soil at Highfield under long‐term grass prior to the establishment of the ley‐arable experiment (Avery & Catt, 1995)

		Sand 2000 to 63 µm	Silt 63 to 2 µm	Clay <2 µm	Soil organic matter	
Depth / m	Horizon	/ % (g 100 g^−1^ soil)				pH (H_2_O) / −log (g [H^+^] L^−1^)
0.00–0.18	Ah	15	59	26	8.2	4.8
0.18–0.30	Eb	15	56	29	6.0	4.9
0.30–0.60+	2Bt(g)	5	32	63	4.5	5.6

### Sampling and processing

In October 2008, a soil sample was taken from a random location in each plot in blocks 1–3 at Highfield, under grass and arable, with a manual gouge auger (diameter 28 mm) to a depth of 0.75 m. To match this replication (*n* = 3), we took one sample from a random location in the Highfield fallow and two samples from the Geescroft fallow (one from each 50‐m half of the 5‐m strip). All samples were then separated into 0–0.15, 0.15–0.30, 0.30–0.45, 0.45–0.60 and 0.60–0.75‐m depth sections. These sections broadly correspond to the Ah (0–0.18 m under grass), Ap (0–0.23 m under arable and fallow), Eb (0.18–0.30 m under grass) and 2Bt(g) horizons (0.30 m onwards) described by Avery & Catt (1995). The depth samples were air‐dried and sieved to < 2 mm. A subsample was finely milled (to < 350 µm) with a Retsch PM 400 planetary ball mill (Retsch GmbH & Co. KG., Haan, Germany) for all analyses. A small subsample was oven‐dried at 105°C for 48 hours to calculate water contents and dry masses. There were 45 samples in total (three treatments × three field replicates × five depth sections) and from each a single subsample was subjected to the analyses.

### Soil analysis

Soil was analysed for SOC and total N content by dry combustion in a Leco TruMac Combustion Analyser (LECO Corp., St Joseph, MI, USA). The pH of the soil at Highfield under the three treatments is 5.1–5.5 (Hirsch *et al.*, 2009) with no carbonates (Avery & Catt, 1995), therefore acid pretreatment was not required. Stable ^13^C and ^15^N isotope values were determined with a SerCon 20‐22 Isotope Ratio Mass Spectrometer (SerCon Ltd, Crewe, UK). By convention, the abundance ratios ^13^C/^12^C and ^15^N/^14^N were expressed as *δ* values (‰) relative to the international Vienna‐Pee Dee Belemnite and atmospheric N standards, respectively. Wheat flour (*δ* ^13^C = −26.41‰, *δ* ^15^N = 4.80‰) calibrated against IAEA‐N‐1 (Iso‐Analytical, Crewe, UK) was used as a reference standard. The instrument error was ± 0.3‰.

The concentrations of long‐chain *n*‐alkanes (C_23_–C_33_) were quantified according to Norris *et al.* (2013). Briefly, a 10‐g sample was Soxhlet‐extracted with (high‐performance liquid chromatography) HPLC‐grade dichloromethane and acetone (9:1) for 24 hours, with C_34_ *n*‐alkane added as an internal standard. The extract was evaporated to dryness and hydrolysed with 0.5 m NaOH (100°C, 1 hour) before neutralization with 1 m HCl and extraction with diethyl ether. The extract was separated into three fractions by silica gel flash‐column chromatography. Elution of the first fraction with hexane gave the aliphatic hydrocarbons. This fraction was re‐dissolved in hexane and analysed with an Agilent 7890A GC fitted with an Agilent HP‐5 column (30 m × 320 µm × 0.25 µm; l × i.d. × film) and a flame ionization detector (Agilent Technologies Inc., Santa Clara, CA, USA). The oven temperature increased from 40°C (held for 1 minute) to 130°C at 20°C minute^−1^, then to 300°C (held for 10 minutes) at 4°C minute^−1^.

### Statistical analysis

All statistical analysis was carried out with GenStat (16th Edition) (VSN International Ltd, Hemel Hempstead, UK). The analysis was non‐trivial because, firstly, the fallow treatment was not part of the ley‐arable experiment and was not replicated in complete blocks. We used residual maximum likelihood (REML) for the following model structures:

(1) $$\text{Fixed}:\;\left(\text{Experiment}/\text{Treatment}\right)\;*\;\text{Depth}\text{,}$$

(2) Random: Experiment×Block/Plot/Depth,

where ‘Experiment’ allocated data according to whether they were derived from either the original ley‐arable long‐term experiment or the complementary bare fallow plots. The fixed model above incorporated the three treatments within the appropriate original experiment before forming the cross‐products with the depth factor (indicated by *). Likewise, the random model confirmed that there was no single block factor for all data, but rather there were separate blocks for the two experiments. It is important to note that we have experiment and depth as separate (though identical) random and fixed factors.

The second non‐trivial aspect of the analysis was that there was no strict independence of samples at different depths from each plot because a single sample was taken and subdivided into depth samples. We may expect correlations between pairs of depths for any property to vary rather than be constant, depending on the distance between them. Therefore we needed to test for autocorrelation with depth in our profile measurements by introducing an autoregressive variance structure into our REML models:

(3) Variance: Experiment×Block×Plot×Depth.

GenStat was unable to test for autocorrelation with the above model because of unsatisfactory estimation of parameters and components going out of bounds. The small number of depth intervals (five) was probably a contributing factor in this. We tried several REML models, but could only obtain a result for the autocorrelation if we set a balanced random model, which suggested that all treatments were in randomized complete blocks, which was not so.

Because we were unable to describe the random and fixed models accurately and to test for autocorrelation with the depth factor simultaneously in the same REML analysis, we adopted a REML‐based parsimonious modelling approach. The crucial difference was that we treated depth in the fixed model as a variable (v) with the value of the midpoint of the interval, and retained depth as a factor (f) in the random model:

(4) $$\text{Fixed}:\;\left(\text{Experiment}/\text{Treatment}\right)\;*\;{\text{Depth}}_{v}\text{,}$$

(5) $$\;\text{Random: Experiment}\;\times \;\text{Block}/\text{Plot}/{\text{Depth}}_{f}\text{.}$$

This formulation accepts that measurements from our five depth intervals are not discrete, but are part of a continuum down the profile. It addresses the likely autocorrelation with depth, and retains the true representation of the field experiment in the random model. We analysed SOC, N, C:N ratio, *δ* ^13^C, *δ* ^15^N and both total odd‐chained and individual *n*‐alkane concentrations with the REML model structures above, and then repeated the analysis with a spline model to determine whether treatment‐specific splines modelled the data as effectively:

(6) $$\text{Spline}:\;{\text{Depth}}_{v}/\text{Treatment}\text{.}$$

To determine whether fitting the spline model improved the model fit significantly, we examined the difference in deviance between the two REML analyses and compared the value with the critical chi‐squared (*χ* ^2^) value at *P* < 0.05 and 2 degrees of freedom (d.f.) (the difference in d.f. between the two REML analyses was 2). Table 2 gives an example of a fixed effects output for SOC from the REML analysis, and shows how the fixed model (4) and its cross‐products expand. We used the GenStat VPREDICT command to derive appropriate treatment‐specific model fits to our data, either parallel linear, non‐parallel linear or spline curve depending on which fixed effect interactions were significant. We used the fitted models to test for significant effects of treatment and depth. All our measured data for SOC, N, C:N ratio, *δ* ^13^C, *δ* ^15^N and total odd‐chained *n*‐alkane concentrations are given in Table S1.

**Table 2:** The residual maximum likelihood (REML) table for soil organic carbon (SOC) (g 100 g^−1^; %) under long‐term grass, arable (1949–) or fallow (1959–) treatments in 2008 with the REML structure for splines outlined in models (4), (5), (6)

Fixed term	Wald statistic	n.d.f.	*F* statistic	d.d.f.	*P*
Experiment	153.1	1	153.1	29.2	< 0.001
Depth_v_	545.2	1	545.2	29.1	< 0.001
Experiment × Treatment	137.8	1	137.8	29.0	< 0.001
Experiment × Depth_v_	100.5	1	100.5	29.1	< 0.001
Experiment × Treatment × Depth_v_	129.0	1	129.0	29.0	< 0.001

**Figure 2:**
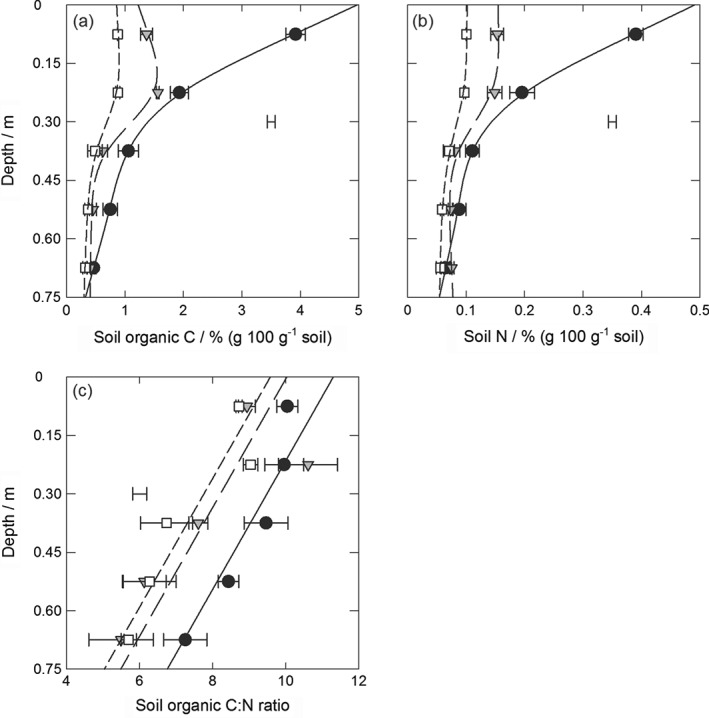
(a) Soil organic carbon (C), (b) nitrogen (N) and (c) organic C:N ratio under long‐term grass (black circles, solid line), arable (1949–; grey triangles, long‐dashed line) and fallow (1959–; white squares, short‐dashed line) treatments in 2008. The data points show the treatment mean and standard error of the mean (n = 3) and the lines derive from residual maximum likelihood (REML) analysis with spline or linear models where appropriate (models (4), (5), (6)). The separate bar shows the average standard error of a difference of means (SED) for the model (see Tables 2 and 3 for statistical analysis).

The table gives the degrees of freedom (d.f.) associated with the numerator (n) and denominator (d), the Wald statistic, the variance ratio (F) statistic and the probability level associated with F (P) to assess experiment and treatment factors, the depth variate (v) and their interactions. Note that the Wald statistic is identical to the F statistic because the numerator d.f. was 1. SOC is shown in Figure 2(a).

## Results

### Soil organic C and N

Figure 2 shows SOC, N and their ratio in the profile and Table 3 summarizes the statistical analysis (see also Table 2 for the REML fixed effects output for SOC). The (experiment/treatment) × depth interaction and treatment‐specific splines were significant for SOC and N (at *P* < 0.001 and < 0.05, respectively). Figure 2 shows that contents of SOC and N decreased significantly in the upper 0.3 m where the treatment changed from grass to arable to fallow (*P* < 0.05). In addition, SOC and N contents remained significantly larger under grass than under the other treatments down to 0.60 and 0.45 m, respectively (*P* < 0.05). The (experiment/treatment) nested factor was significant for the C:N ratio (*P* = 0.002) where soil under grass had a significantly larger C:N ratio (*P* < 0.05), but the interaction with depth was not significant (*P* = 0.122). The C:N ratio decreased with depth (*P* < 0.001). Splines were not significant (*P* > 0.05).

**Table 3:** Residual maximum likelihood (REML) analysis of soil organic carbon (SOC), nitrogen (N), C:N ratio, the stable isotope ratios δ ^13^C and δ ^15^N, and total odd‐chained n‐alkane concentration (C_23_–C_33_) of soil under long‐term grass, arable (1949–) or fallow (1959–) treatments in 2008 to test for effects of the experiment (E) and treatment (T) factors and the depth (D_v_) variate

Property	Spline model	*σ* ^2^	SE	Deviance	d.f	Significant improvement with spline?	Significant interaction	*F* statistic	d.d.f	*P*	SED
SOC / % (g 100 g^−1^)	None	0.144	0.033	−1.21	35	–	–	–	–	–	–
	D_v_ / T	0.030	0.008	−39.46	33	Yes	E × T × D_v_	128.99	29.0	< 0.001	0.135
N / % (g 100 g^−1^)	None	0.0014	0.0003	−178.06	35	–	–	–	–	–	–
	D_v_ / T	0.0003	0.0001	−218.47	33	Yes	E × T × D_v_	153.03	23.8	< 0.001	0.013
C:N ratio	None	1.092	0.250	75.80	35	–	E / T	11.17	38.0	0.002	0.381
	D_v_ / T	0.866	0.208	72.46	33	No	–	–	–	–	–
*δ* ^13^C / ‰	None	0.151	0.038	9.56	35	–	E × T × D_v_	9.75	31.9	0.004	0.279
	D_v_ / T	0.118	0.032	6.31	33	No	–	–	–	–	–
*δ* ^15^N / ‰	None	0.602	0.138	53.16	35	–	E × T × D_v_	7.60	38.0	0.009	0.367
	D_v_ / T	0.560	0.151	52.63	33	No	–	–	–	–	–
*n*‐alkane / µg g^−1^	None	1.633	0.402	97.57	36	–	–	–	–	–	–
	D_v_ / T	0.819	0.225	84.11	34	Yes	E × T × D_v_	4.07	26.6	0.054	0.790

**Figure 3:**
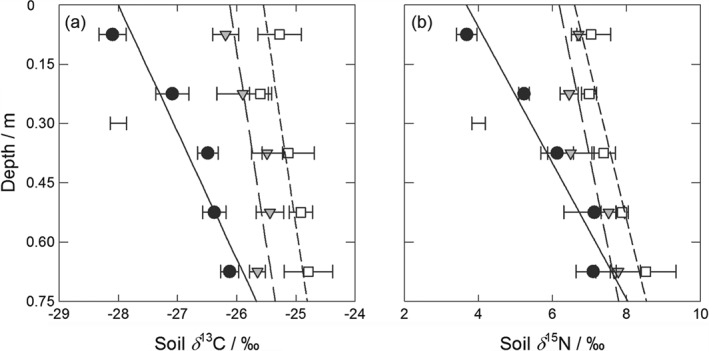
(a) Soil stable δ ^13^C and (b) δ ^15^N under long‐term grass (black circles, solid line), arable (1949–; grey triangles, long‐dashed line) and fallow (1959–; white squares, short‐dashed line) treatments in 2008. The data points show the treatment mean and standard error of the mean (n = 3) and the lines derive from residual maximum likelihood (REML) analysis with linear models (models (4) and (5)). The separate bar shows the average standard error of a difference of means (SED) for the model (see Table 3 for statistical analysis).

**Figure 4:**
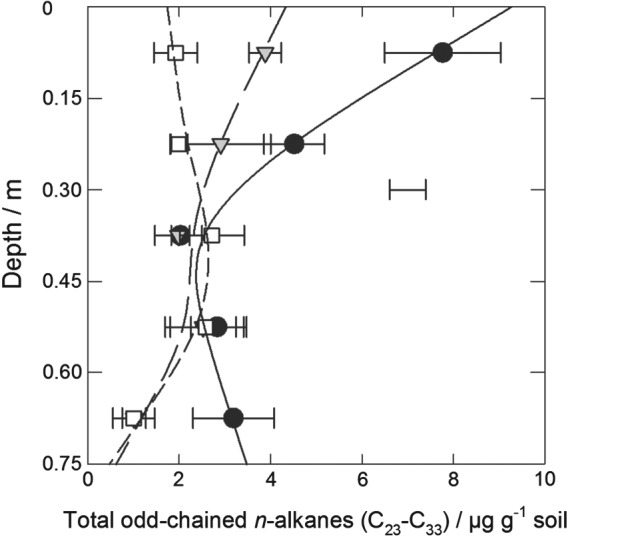
Concentration of total odd long‐chain n‐alkanes (from C_23_ to C_33_) in soil under long‐term grass (black circles, solid line), arable (1949–; grey triangles, long‐dashed line) and fallow (1959–; white squares, short‐dashed line) treatments in 2008. The data points show the treatment mean and standard error of the mean (n = 3) and the lines derive from residual maximum likelihood (REML) analysis with spline models (models (4), (5), (6)). The separate bar shows the average standard error of a difference of means (SED) for the model (see Table 3 for statistical analysis).

The estimate and standard error (SE) of the residual variance (σ ^2^), the deviance (−2 × log‐likelihood) and degrees of freedom (d.f.) are given for REML analyses without and with a fitted spline model, and the significance of the change in deviance was checked with reference to the critical χ ^2^ value. The variance ratio (F) statistic with the denominator d.f. (d.d.f.), the probability level associated with the variance ratio (P) and the average standard error of a difference of means (SED) are given for the interaction of factors and the variate. Note that the Wald statistic is identical to the *F* statistic because the numerator d.f. was 1. The REML structures are outlined in models (4), (5), (6) and the appropriate models are shown in Figures 2, 3, 4.

### Stable ^13^C and ^15^N isotopes

Stable isotope ratios of *δ* ^13^C and *δ* ^15^N are shown in Figure 3, and Table 3 shows that the (experiment/treatment) × depth interactions were again significant (*P* = 0.004 and 0.009, respectively). Splines did not result in a significant improvement over the linear models for both *δ* ^13^C and *δ* ^15^N (*P* > 0.05). At each depth both *δ* ^13^C and *δ* ^15^N increased in the order grass < arable < fallow soil. For *δ* ^13^C and *δ* ^15^N, the differences between grass and fallow soil were significant at all depths, and significant between grass and arable soil to 0.60 m depth for *δ* ^13^C and to 0.45 m for *δ* ^15^N (*P* < 0.05).

### Long‐chain n‐alkanes

Concentrations of odd‐chained *n*‐alkanes were much greater than those of even‐chained *n*‐alkanes (see Table S2). It was for the *n*‐alkanes C_27_, C_29_ and C_31_ only that both spline curves gave significantly improved model fits (*P* < 0.05) and that the (experiment/treatment) × depth interactions were significant (*P* = 0.014, 0.051 and 0.025, respectively) (see Table 4). In the upper 0.15 m of the soil, there were larger concentrations of these three *n*‐alkanes under grass than under arable and fallow (*P* < 0.05). The same was also true for C_29_ and C_31_ at 0.60–0.75‐m depth (*P* < 0.05). In addition, there were larger concentrations of C_29_ and C_31_ at 0.15–0.30‐m depth for soil under grass than fallow (*P* < 0.05). Total odd‐chained *n*‐alkane concentrations (C_23_–C_33_) are shown in Figure 4, and the results of the analysis are given in Table 3. The (experiment/treatment) × depth interaction (*P* = 0.054) mirrored that described for C_29_ above; total concentrations in soil under grass were greater than those under either fallow (0.15–0.30‐m depth) or both fallow and arable (0–0.15 and 0.60–0.75‐m depths; *P* < 0.05).

**Table 4:** Concentration (upper table) and residual maximum likelihood (REML) analysis (lower table) of the long‐chain n‐alkanes C_27_, C_29_ and C_31_ of soil under long‐term grass, arable (1949–) or fallow (1959–) treatments in 2008, where the interaction of experiment (E) and treatment (T) factors and the depth (D_v_) variate was significant with a fitted D_v_/T spline model

	*n*‐alkane								
	C_27_			C_29_			C_31_		
	Grass	Arable	Fallow	Grass	Arable	Fallow	Grass	Arable	Fallow
Soil depth / m	Concentration (mean; *n* = 3) / µg g^−1^ soil								
0–0.15	1.20	0.59	0.42	2.11	0.93	0.50	2.62	1.15	0.45
0.15–0.30	0.75	0.42	0.38	1.06	0.77	0.45	1.32	0.90	0.42
0.30–0.45	0.48	0.41	0.67	0.45	0.42	0.58	0.44	0.43	0.48
0.45–0.60	0.57	0.61	0.64	0.61	0.51	0.54	0.60	0.43	0.42
0.60–0.75	0.48	0.24	0.20	0.72	0.16	0.15	0.91	0.23	0.14

REML statistic	REML values		
*σ* ^2^	0.026	0.073	0.089
SE	0.007	0.020	0.024
Deviance	−47.03	−13.63	−6.31
d.f.	34	34	34
*F* statistic	6.87	4.15	5.65
d.d.f.	26.3	27.3	27.3
*P*	0.014	0.051	0.025
SED	0.149	0.215	0.227

The estimate and standard error (SE) of the residual variance (σ ^2^), the deviance (−2 × log‐likelihood) and degrees of freedom (d.f.) are given with the variance ratio (F) statistic with the denominator d.f. (d.d.f.), the probability level associated with the variance ratio (P), and the average standard error of a difference of means (SED) for the E·T·D_v_ interaction. Note that the Wald statistic is identical to the F statistic because the numerator d.f. was 1. The REML structures are outlined in models (4), (5), (6).

## Discussion

### Soil organic C and N

In the top 0.15 m, SOC and N concentration losses in the former grass soil were 65 and 61% since the change to arable, and 78 and 74% since the change to fallow, respectively. This is similar to that reported for the same soil types by Wu *et al.* (2012). With bulk density data measured separately in the same plots at a similar time (0.99 g cm^−3^ for grass, 1.30–1.45 g cm^−3^ for arable and fallow), SOC and N stocks determined in the top 0.15 m were 58 and 5.8 Mg ha^−1^ under grass, 30 and 3.3 Mg ha^−1^ under arable and 17 and 2.0 Mg ha^−1^ under fallow, respectively. Equivalent stock losses of SOC and N were therefore 49 and 43% under arable and 70 and 66% under fallow, respectively. These calculations probably represent an upper estimate of the losses because there is evidence that there was a marginal increase in SOC in the upper 0.23 m under the long‐term grass treatment at Highfield between 1949 and 1960, with little change thereafter (Johnston *et al.*, 2009). Nevertheless, these losses are much greater than the 35% loss of SOC reported in the top 0.23 m in the same arable soil during the first 20 years after the change from grass (Johnston *et al.*, 2009). Furthermore, Poeplau *et al.* (2011), in a review of grass‐to‐arable changes under temperate climates more generally, reported a loss of 36% of SOC over the same period, which suggests continuous loss from cultivation. It is widely reported that it is easier to lose SOC than to gain it (Don *et al.*, 2009; Bell *et al.*, 2011). Johnston *et al.* (2009) demonstrated precisely this in their comparison of the effects on SOC of grass‐to‐arable and arable‐to‐grass experiments at Rothamsted.

There are few reports of the changes in processes in subsoils after land‐use change (e.g. Jenkinson *et al.*, 2008; Beniston *et al.*, 2014). Some suggest that management affects only the distribution rather than the overall amount of SOC (Don *et al.*, 2009). We observed the homogenizing effect of cultivation on SOC in the upper 0.30 m under arable and fallow and the contrasting gradual decline under grass. Nevertheless, we detected significant effects of land‐use change on both SOC and N to 0.6 m depth between grass and fallow treatments (Table 3; Figure 1), and almost twice as much SOC content under grass than under arable averaged over the 0.75‐m profile depth. This is similar to that reported in soil profiles in Scotland (Chapman *et al.*, 2013). Arable and fallow treatments resulted in losses of 41 and 52% of SOC and 22 and 35% of N concentration, respectively, at the 0.30–0.60‐m depth by 2008, compared with soil under grass. The equivalent stock losses (with a measured bulk density of 1.20 g cm^−3^ for all treatments) were 33 and 59% of SOC and 13 and 45% of N, respectively. Although we have no historical data for Highfield to verify equilibrium subsoil conditions since 1949 under grass, data from the Park Grass experiment at Rothamsted show little or only marginal change in SOC and N below 0.23 m over this period (Jenkinson *et al.*, 2008; Gregory *et al.*, 2014). We found that SOC and N were closely linked, but that the C:N ratio decreased with depth. This is commonly ascribed to increased ammonium‐N fixation at depth, particularly in clay subsoil (Jenkinson *et al.*, 2008; Rumpel & Kögel‐Knabner, 2011). Of greater interest, we found differences, albeit insignificant, between the C:N ratios of the different treatments down the soil profile. The C:N ratio under arable was greatest at 0.15–0.30‐m depth. We speculate that this was because of the incorporation of stubble (with a much larger C:N ratio) in the cultivated layer (upper 0.23 m) from the wheat crop following annual harvests.

Harvesting reduces plant residue inputs (i.e. wheat grain and straw) except for the incorporation of stubble, and cultivation encourages microbial oxidation of SOC in the topsoil of arable systems. These factors are exacerbated under fallow management where there is no plant input. At Highfield, for example, the soil has been cultivated annually with no plant input since 1959 under fallow. Grasslands have a more extensive root system than arable crops, larger inputs of C, larger faunal populations (Hirsch *et al.*, 2009) and a greater potential for C movement in dissolved forms (Baisden *et al.*, 2002; Kindler *et al.*, 2011). The measured C:N ratio in the subsoil under grass was more similar to that in the topsoil than for the other treatments, which supports the maintained delivery of fresh organic material under grass. Annual incorporation of wheat stubble in the upper 0.23‐m cultivated layer probably explains the increased SOC and C:N ratio measured at 0.15–0.30 m compared with the uppermost layer under arable.

### Stable ^13^C and ^15^N isotopes

The observed general increase in both *δ* ^13^C and *δ* ^15^N with depth is common (Fontaine *et al.*, 2007; Jenkinson *et al.*, 2008). Rayleigh distillation (the kinetic fractionation against the heavier isotope during microbial metabolism) causes ^13^C‐enrichment of ‘older’ SOC in most subsoil (Jenkinson *et al.*, 2008; Beniston *et al.*, 2014). Several variables control soil *δ* ^13^C, including changes in atmospheric CO_2_ (Randerson *et al.*, 1999), isotopic discrimination during decomposition (Mariotti & Balesdent, 1990), and both inter‐ and intra‐specific variation in vegetation input signatures caused by physiological stresses (Dungait *et al.*, 2008, 2010; Köhler *et al.*, 2010). Microbial processing of old SOC with little or no new inputs over decades under arable and fallow, compared with soil under grass that continued to receive fresh inputs with a near‐unchanged isotopic composition, probably caused the differences we observed between the treatments. Larger *δ* ^13^C values for topsoil and subsoil under arable than under grass have been reported previously (Jenkinson *et al.*, 2008; Dixon *et al.*, 2010). Mean (*n* = 6) *δ* ^13^C values for grass and wheat plant material (leaf, straw and root) from Highfield were significantly different (*P* = 0.008, SED = 0.289, d.f. = 2) at −30.7 and −27.4‰, respectively. The decrease in the *δ* ^13^C value of atmospheric CO_2_ over time (Zhao *et al.*, 2001) might partly explain the greater soil *δ* ^13^C we observed under fallow, where all SOC derives strictly from before 1959. There were no significant differences in *δ* ^15^N between grass and wheat plant material (*P* = 0.428, SED = 0.748, d.f. = 2), with averages (*n* = 6) of 1.7 and 1.0‰, respectively. The source of N in soil is multifarious and subject to a wide range of transformations that affect *δ* ^15^N. Loss of ^14^N during denitrification can increase soil *δ* ^15^N (Dixon *et al.*, 2010); the potential for compaction, increased water‐filled pore space and denitrification are greater under arable and fallow systems than under grass. Ponding has been observed on the fallow plots during wet winters, which encourages denitrification.

### Long‐chain n‐alkanes

Long‐chain *n*‐alkanes from plant waxes have non‐functionalized aliphatic structures that are considered to be resistant to microbial decomposition (Dungait *et al.*, 2012) with decadal residence times (Amelung *et al.*, 2008). The most abundant *n*‐alkanes were C_27_, C_29_ and C_31_; the latter two are described as grass biomarkers (van Bergen *et al.*, 1997; Norris *et al.*, 2013). The *n*‐alkane concentrations in the 0–0.3‐m depth were significantly larger under grass than under arable and fallow (Tables 3, 4; Figure 3), which reflects leaf and litter inputs. Nevertheless, the wheat crop provided some *n*‐alkanes to the topsoil. The concentration in the 0–0.15 and 0.15–0.30‐m depths under fallow was 27 and 52% only of that in soil under grass, respectively, after 49 years with no fresh input. We found significant treatment differences in *n*‐alkane concentrations at the 0.60–0.75‐m depth compared with the overlying depth (Tables 3, 4; Figure 3): concentrations decreased under arable and fallow, but increased under grass. Radiocarbon dating of similar soil at Rothamsted by Jenkinson *et al.* (2008) suggested that fresh plant‐derived C finds its way down the profile, which suggests a link between surface vegetation and subsoil SOC. Long‐chain *n*‐alkanes are unlikely to be transported by water because they are extremely non‐polar. Therefore, our observations in subsoil under grass might indicate input by plant roots or bioturbation by anecic earthworms, both of which are likely to be more prevalent under grass. There might also have been preferential turnover of *n*‐alkanes at depth in soil under arable and fallow, although the mechanism for this is unknown. Wu *et al.* (2012) observed more substrate C mineralization in topsoil under fallow than grass at Highfield. This suggests that mineralization can occur where C is limited, such as in the subsoil, although this was based on laboratory experiments on disturbed soil. Nevertheless, the turnover of compounds usually assumed to be resistant in the subsoil on decadal time‐scales indicates that land‐use change might affect quite stable SOC pools in the whole soil profile.

## Conclusions

The legacy of converting permanent grassland to arable and fallow management 59 and 49 years before, respectively, on SOC and N contents was evident not only in the top 0.15 m, where up to 65% (arable) and 78% (fallow) of both SOC and N were lost, but also in the subsoil at 0.30–0.60 m, where up to 41% (arable) and 59% (fallow) of both were lost. We are confident of our estimate of losses in the subsoil, but those given above for the upper 0.15 m probably represent an upper estimate from evidence that there was a marginal increase in SOC under long‐term grass in the first decade of the experiment. Isotopic and biomarker analyses indicate that the quantity and quality of compounds returned to the soil were reduced, with a reduction in the delivery of colloid‐bound or dissolved SOC and N, and increased turnover of existing SOC and N in arable and fallow systems. The effect of cultivation under arable and fallow systems was evident in the greater homogeneity of the various properties measured in the upper two depths and in the incorporation of wheat stubble under the former. The soil under undisturbed grass was heterogeneous and showed more gradual change in such properties with depth. Significant differences in the subsoil suggest that it is biologically active and more affected by land use than was probably envisaged, at least on decadal time‐scales. There is a clear need, therefore, to monitor, model and predict changes in soil properties in the long term not just near the surface but for the whole soil profile with the use of controlled experiments such as that described here. This will increase our ability to manage soil sustainably and deliver both food security and other ecosystem services, especially climate change mitigation through C sequestration.

## Supporting information


**Table S1.** Soil organic carbon (SOC), nitrogen (N), C : N ratio, the stable isotope ratios δ^13^C and δ^15^N, and total odd‐chained n‐alkane concentration (C_23_–C_33_) of soil under long‐term grass, arable (1949–) or fallow (1959–) treatments in 2008.

**Table S2.** Concentration and residual maximum likelihood (REML) analysis of long‐chain n‐alkanes (from C_23_ to C_33_) of soil under long‐term grass, arable (1949–) or fallow (1959–) treatments in 2008.
