# Supplementary material for: Long‐term management changes topsoil and subsoil organic carbon and nitrogen dynamics in a temperate agricultural system
Source: Eur J Soil Sci. 2016 Jul 15;67(4):421–30. doi: 10.1111/ejss.12359 (PMC4950136; doi:10.1111/ejss.12359)
Supplement: Supplementary file 1 — Table S1. Soil organic carbon (SOC), nitrogen (N), C : N ratio, the stable isotope ratios δ13C and δ15N, and total odd‐chained n‐alkane concentration (C23–C33) of soil under long‐term grass, arable (1949–) or fallow (1959–) treatments in 2008. [file EJSS-67-421-s002.docx]

**Table S1**Soil organic carbon (SOC), nitrogen (N), C:N ratio, the stable isotope ratios *δ*^13^C and *δ*^15^N and total odd-chained *n*-alkane concentration (C_23_–C_33_) of soil under long-term grass, arable (1949–) or fallow (1959–) treatments in 2008. Missing values are indicated by –.

| Field | Experiment | Treatment | Block | Plot | Depth | SOC | N | CN | *δ*^13^C | *δ*^15^N | *n*-alkanes |
| --- | --- | --- | --- | --- | --- | --- | --- | --- | --- | --- | --- |
|  |  |  |  |  | / m | / % (g 100 g^-1^ soil) | | ratio | / ‰ | / ‰ | / µg g^-1^ soil |
| Highfield | Ley-arable | Grass | 1 | 05/06 | 0–0.15 | 4.076 | 0.413 | 9.86 | −27.75 | 3.94 | 8.480 |
|  |  |  |  |  | 0.15–0.30 | 1.792 | 0.163 | 11.02 | −26.87 | 5.38 | 5.194 |
|  |  |  |  |  | 0.30–0.45 | 1.399 | 0.133 | 10.51 | −26.82 | 5.48 | 2.328 |
|  |  |  |  |  | 0.45–0.60 | 0.706 | 0.084 | 8.39 | −26.02 | 6.55 | 3.924 |
|  |  |  |  |  | 0.60–0.75 | 0.455 | 0.075 | 6.07 | −25.83 | 8.01 | 1.709 |
|  |  |  | 2 | 13/14 | 0–0.15 | 4.092 | 0.386 | 10.61 | −28.01 | 3.13 | 9.522 |
|  |  |  |  |  | 0.15–0.30 | 1.762 | 0.189 | 9.32 | −26.76 | 5.37 | 5.164 |
|  |  |  |  |  | 0.30–0.45 | 0.956 | 0.101 | 9.43 | −26.41 | 5.93 | 2.110 |
|  |  |  |  |  | 0.45–0.60 | 0.558 | 0.070 | 7.97 | −26.42 | 8.75 | 2.604 |
|  |  |  |  |  | 0.60–0.75 | 0.394 | 0.050 | 7.95 | −26.18 | 6.64 | 4.767 |
|  |  |  | 3 | 31/32 | 0–0.15 | 3.587 | 0.371 | 9.66 | −28.53 | 3.96 | 5.308 |
|  |  |  |  |  | 0.15–0.30 | 2.237 | 0.235 | 9.53 | −27.65 | 4.94 | 3.200 |
|  |  |  |  |  | 0.30–0.45 | 0.817 | 0.097 | 8.43 | −26.23 | 6.96 | 1.646 |
|  |  |  |  |  | 0.45–0.60 | 0.980 | 0.110 | 8.94 | −26.69 | 6.07 | 1.982 |
|  |  |  |  |  | 0.60–0.75 | 0.540 | 0.070 | 7.73 | −26.35 | 6.64 | 3.091 |
| Highfield | Ley-arable | Arable | 1 | 09/10 | 0–0.15 | 1.405 | 0.165 | 8.53 | −25.78 | 6.70 | 4.568 |
|  |  |  |  |  | 0.15–0.30 | 1.557 | 0.159 | 9.79 | −26.11 | 6.23 | 4.257 |
|  |  |  |  |  | 0.30–0.45 | 0.729 | 0.092 | 7.96 | −25.62 | 7.26 | 2.712 |
|  |  |  |  |  | 0.45–0.60 | 0.518 | 0.081 | 6.37 | −25.77 | 7.21 | 3.657 |
|  |  |  |  |  | 0.60–0.75 | 0.345 | 0.069 | 5.03 | −25.39 | 7.27 | 1.310 |
|  |  |  | 2 | 19/20 | 0–0.15 | 1.524 | 0.164 | 9.32 | −26.27 | 6.62 | 3.421 |
|  |  |  |  |  | 0.15–0.30 | 1.606 | 0.164 | 9.81 | −26.53 | 6.18 | 0.750 |
|  |  |  |  |  | 0.30–0.45 | 0.669 | 0.086 | 7.75 | −25.86 | 6.96 | 2.251 |
|  |  |  |  |  | 0.45–0.60 | 0.504 | 0.072 | 7.01 | −25.57 | 7.44 | 2.723 |
|  |  |  |  |  | 0.60–0.75 | 0.541 | 0.075 | 7.19 | −25.82 | 7.08 | 0.495 |
|  |  |  | 3 | 27/28 | 0–0.15 | 1.176 | 0.131 | 8.96 | −26.52 | 6.78 | 3.663 |
|  |  |  |  |  | 0.15–0.30 | 1.519 | 0.124 | 12.23 | −25.07 | 6.93 | 3.727 |
|  |  |  |  |  | 0.30–0.45 | 0.459 | 0.065 | 7.11 | −24.98 | 5.25 | 0.987 |
|  |  |  |  |  | 0.45–0.60 | 0.334 | 0.067 | 4.99 | −25.00 | 7.90 | 1.197 |
|  |  |  |  |  | 0.60–0.75 | 0.347 | 0.082 | 4.24 | −25.76 | 8.98 | 1.231 |
| Geescroft | Bare fallow | Fallow | 1 | 1 | 0–0.15 | 0.866 | 0.100 | 8.62 | −24.81 | 7.73 | 2.858 |
|  |  |  |  |  | 0.15–0.30 | 0.875 | 0.097 | 9.02 | −25.59 | 7.21 | 2.378 |
|  |  |  |  |  | 0.30–0.45 | 0.367 | 0.061 | 6.06 | −24.69 | 7.64 | 4.120 |
|  |  |  |  |  | 0.45–0.60 | 0.430 | 0.061 | 7.00 | −25.12 | 7.71 | 4.145 |
|  |  |  |  |  | 0.60–0.75 | 0.248 | 0.042 | 5.89 | −24.92 | 7.66 | 1.827 |
|  |  |  | 2 | 2 | 0–0.15 | 0.876 | 0.101 | 8.65 | −25.03 | 7.39 | 1.262 |
|  |  |  |  |  | 0.15–0.30 | 0.936 | 0.100 | 9.39 | −25.28 | 7.18 | 1.758 |
|  |  |  |  |  | 0.30–0.45 | 0.365 | 0.061 | 5.99 | −24.69 | 7.75 | 2.169 |
|  |  |  |  |  | 0.45–0.60 | 0.310 | 0.056 | 5.55 | −24.73 | 8.04 | 2.555 |
|  |  |  |  |  | 0.60–0.75 | 0.319 | 0.060 | 5.29 | −24.02 | 10.14 | 0.934 |
| Highfield | Bare fallow | Fallow | 3 | 3 | 0–0.15 | 0.879 | 0.099 | 8.88 | −26.01 | 6.00 | 1.653 |
|  |  |  |  |  | 0.15–0.30 | 0.815 | 0.094 | 8.70 | −25.93 | 6.58 | 1.846 |
|  |  |  |  |  | 0.30–0.45 | 0.732 | 0.090 | 8.16 | −26.02 | 6.76 | 1.857 |
|  |  |  |  |  | 0.45–0.60 | – | – | – | – | – | 1.059 |
|  |  |  |  |  | 0.60–0.75 | 0.381 | 0.064 | 5.93 | −25.42 | 7.77 | 0.256 |
