# Supplementary material for: Long‐term management changes topsoil and subsoil organic carbon and nitrogen dynamics in a temperate agricultural system
Source: Eur J Soil Sci. 2016 Jul 15;67(4):421–30. doi: 10.1111/ejss.12359 (PMC4950136; doi:10.1111/ejss.12359)
Supplement: Supplementary file 2 — Table S2. Concentration and residual maximum likelihood (REML) analysis of long‐chain n‐alkanes (from C23 to C33) of soil under long‐term grass, arable (1949–) or fallow (1959–) treatments in 2008. [file EJSS-67-421-s001.docx]

**Table S2**Concentration (upper table) and residual maximum likelihood (REML) analysis (lower table) of long-chain *n*-alkanes (from C_23_ to C_33_) of soil from under long-term grass, arable (1949–) or fallow (1959–) treatments in 2008, testing for effects of the experiment (E) and treatment (T) factors and the depth (D_v_) variate. Note that some *n*-alkanes were not detected (n.d.).

|  |  | *n*-alkane | | | | | | | | | | |
| --- | --- | --- | --- | --- | --- | --- | --- | --- | --- | --- | --- | --- |
|  |  | C_23_ | C_24_ | C_25_ | C_26_ | C_27_ | C_28_ | C_29_ | C_30_ | C_31_ | C_32_ | C_33_ |
| Treatment | Soil depth / m | Concentration (mean ± standard error of the mean; *n*=3) / µg g^-1^ soil | | | | | | | | | | |
| Grass | 0–0.15 | 0.23 ±0.07 | n.d. | 0.59 ±0.08 | n.d. | 1.20 ±0.24 | n.d. | 2.11 ±0.49 | n.d. | 2.62 ±0.48 | n.d. | 1.10 ±0.17 |
|  | 0.15–0.30 | 0.17 ±0.03 | 0.30 ±0.05 | 0.51 ±0.09 | 0.52 ±0.10 | 0.75 ±0.15 | 0.56 ±0.13 | 1.06 ±0.17 | 0.51 ±0.13 | 1.32 ±0.15 | 0.34 ±0.10 | 0.71 ±0.10 |
|  | 0.30–0.45 | 0.09 ±0.01 | 0.21 ±0.02 | 0.33 ±0.06 | 0.40 ±0.08 | 0.48 ±0.08 | 0.34 ±0.06 | 0.45 ±0.04 | 0.23 ±0.03 | 0.44 ±0.02 | 0.13 ±0.02 | 0.24 ±0.01 |
|  | 0.45–0.60 | 0.17 ±0.04 | 0.36 ±0.08 | 0.51 ±0.10 | 0.50 ±0.10 | 0.57 ±0.09 | 0.44 ±0.09 | 0.61 ±0.12 | 0.39 ±0.09 | 0.60 ±0.14 | 0.26 ±0.08 | 0.37 ±0.10 |
|  | 0.60–0.75 | 0.11 ±0.01 | n.d. | 0.33 ±0.06 | n.d. | 0.48 ±0.04 | n.d. | 0.72 ±0.20 | n.d. | 0.91 ±0.32 | n.d. | 0.63 ±0.33 |
| Arable | 0–0.15 | 0.25 ±0.13 | n.d. | 0.36 ±0.06 | n.d. | 0.59 ±0.12 | n.d. | 0.93 ±0.10 | n.d. | 1.15 ±0.11 | n.d. | 0.61 ±0.18 |
|  | 0.15–0.30 | 0.12 ±0.04 | 0.20 ±0.08 | 0.30 ±0.11 | 0.22 ±0.07 | 0.42 ±0.15 | 0.22 ±0.08 | 0.77 ±0.30 | 0.29 ±0.12 | 0.90 ±0.34 | 0.18 ±0.07 | 0.40 ±0.14 |
|  | 0.30–0.45 | 0.13 ±0.02 | 0.27 ±0.07 | 0.37 ±0.10 | 0.33 ±0.10 | 0.41 ±0.13 | 0.26 ±0.08 | 0.42 ±0.11 | 0.23 ±0.05 | 0.43 ±0.11 | 0.13 ±0.02 | 0.23 ±0.05 |
|  | 0.45–0.60 | 0.17 ±0.04 | 0.34 ±0.09 | 0.54 ±0.13 | 0.56 ±0.09 | 0.61 ±0.10 | 0.45 ±0.08 | 0.51 ±0.13 | 0.38 ±0.12 | 0.43 ±0.14 | 0.37 ±0.11 | 0.42 ±0.14 |
|  | 0.60–0.75 | 0.12 ±0.08 | n.d. | 0.16 ±0.08 | n.d. | 0.24 ±0.09 | n.d. | 0.16 ±0.08 | n.d. | 0.23 ±0.05 | n.d. | 0.14 ±0.01 |
| Fallow | 0–0.15 | 0.28 ±0.07 | n.d. | 0.26 ±0.10 | n.d. | 0.42 ±0.13 | n.d. | 0.50 ±0.13 | n.d. | 0.45 ±0.10 | n.d. | 0.19 ±0.04 |
|  | 0.15–0.30 | 0.13 ±0.01 | 0.27 ±0.03 | 0.36 ±0.03 | 0.26 ±0.02 | 0.38 ±0.04 | 0.22 ±0.02 | 0.45 ±0.06 | 0.28 ±0.05 | 0.42 ±0.04 | 0.16 ±0.01 | 0.25 ±0.01 |
|  | 0.30–0.45 | 0.16 ±0.04 | 0.35 ±0.07 | 0.55 ±0.13 | 0.55 ±0.15 | 0.67 ±0.19 | 0.45 ±0.13 | 0.58 ±0.17 | 0.43 ±0.14 | 0.48 ±0.14 | 0.23 ±0.07 | 0.28 ±0.08 |
|  | 0.45–0.60 | 0.20 ±0.07 | 0.39 ±0.08 | 0.55 ±0.22 | 0.55 ±0.20 | 0.64 ±0.23 | 0.45 ±0.16 | 0.54 ±0.19 | 0.45 ±0.15 | 0.42 ±0.13 | 0.24 ±0.07 | 0.24 ±0.06 |
|  | 0.60–0.75 | 0.34 ±0.12 | n.d. | 0.16 ±0.08 | n.d. | 0.20 ±0.10 | n.d. | 0.15 ±0.06 | n.d. | 0.14 ±0.04 | n.d. | 0.13 ±0.05 |
|  |  |  |  |  |  |  |  |  |  |  |  |  |
| Spline | REML statistics | Values | | | | | | | | | | |
| None | *σ*^2^ | 0.006 | 0.008 | 0.034 | 0.021 | 0.049 | 0.018 | 0.134 | 0.022 | 0.202 | 0.013 | 0.061 |
|  | SE | 0.002 | 0.003 | 0.008 | 0.007 | 0.012 | 0.006 | 0.033 | 0.008 | 0.050 | 0.005 | 0.015 |
|  | Deviance | −100.55 | −47.06 | −51.47 | −25.21 | −37.47 | −28.90 | −1.66 | −24.97 | 11.25 | −35.95 | −32.66 |
|  | df | 32 | 18 | 35 | 18 | 36 | 18 | 36 | 18 | 36 | 17 | 35 |
| D_v_ / T | *σ*^2^ | 0.005 | 0.007 | 0.017 | 0.021 | 0.026 | 0.016 | 0.073 | 0.017 | 0.089 | 0.007 | 0.040 |
|  | SE. | 0.001 | 0.003 | 0.005 | 0.008 | 0.007 | 0.006 | 0.020 | 0.007 | 0.024 | 0.003 | 0.011 |
|  | Deviance | −105.04 | −47.21 | −62.78 | −25.25 | −47.03 | −29.52 | −13.63 | −26.27 | −6.31 | −39.71 | −40.27 |
|  | df | 30 | 16 | 33 | 16 | 34 | 16 | 34 | 16 | 34 | 15 | 33 |
| Significant improvement with spline? | | No | No | Yes | No | Yes | No | Yes | No | Yes | No | Yes |
|  | |  |  |  |  |  |  |  |  |  |  |  |
|  |  | Values for E × T × D_v_ interaction | | | | | | | | | | |
|  | *F* statistic | 0.04 | 0.84 | 1.26 | 4.48 | 6.87 | 5.11 | 4.15 | 1.53 | 5.65 | 3.09 | 0.28 |
|  | d.df | 29.2 | 15.0 | 25.2 | 16.9 | 26.3 | 16.9 | 27.3 | 15.0 | 27.3 | 14.9 | 27.2 |
|  | *P* | 0.836 | 0.374 | 0.272 | 0.050 | 0.014 | 0.037 | 0.051 | 0.236 | 0.025 | 0.099 | 0.598 |
|  | SED | 0.056 | 0.089 | 0.117 | 0.150 | 0.149 | 0.137 | 0.215 | 0.150 | 0.227 | 0.112 | 0.138 |

The estimate and standard error (SE) of the residual variance (*σ*^2^), the deviance (–2 × log-likelihood) and degrees of freedom (df) are given for REML analyses without and with a spline model, and the significance of the change in deviance was checked with reference to the critical *χ*^2^ value. The variance ratio (*F*) statistic with the denominator df (d.df), the probability level associated with the variance ratio (*P*), and the average standard error of a difference of means (SED) are then given for the E · T · D_v_ interaction. Note that the Wald statistic was identical to the *F* statistic because the numerator df was 1. The REML structures are outlined in models (4), (5) and (6).
